# A 1:1 flavone cocrystal with cyclic trimeric perfluoro-*o*-phenyl­enemercury

**DOI:** 10.1107/S2056989024005346

**Published:** 2024-06-14

**Authors:** Egor M. Novikov, Raúl Castañeda, Marina S. Fonari, Tatiana V. Timofeeva

**Affiliations:** a Department of Chemistry New Mexico Highlands University,Las Vegas New Mexico 87701 USA; b Institute of Applied Physics, Moldova State University, Academy str., 5 MD2028, Chisinau, Moldova; Universidad de Los Andes Mérida, Venezuela

**Keywords:** cocrystal, flavone, cyclic trimeric perfluoro-*o*-phenyl­enemercury, crystal structure, weak inter­actions

## Abstract

The title compound, systematic name tris­(μ_2_-perfluoro-*o*-phenyl­ene)(μ_2_-3-phenyl-4*H*-chromen-4-one)-*triangulo*-trimercury, [Hg_3_(C_6_F_4_)_3_(C_15_H_10_O_2_)], crystallizes in the monoclinic *P*2_1_/*n* space group with one flavone (FLA) and one cyclic trimeric perfluoro-*o*-phenyl­enemercury (TPPM) mol­ecule per asymmetric unit. The FLA mol­ecule is located on one face of the TPPM acceptor and is linked in an asymmetric coordination of its carbonyl oxygen atom with two Hg centers of the TPPM macrocycle. The angular-shaped complexes pack in zigzag chains where they stack *via* two alternating TPPM–TPPM and FLA–FLA stacking patterns. The distance between the mean planes of the neighboring TPPM macrocycles in the stack is 3.445 (2) Å, and that between the benzo-γ-pyrone moieties of FLA is 3.328 (2) Å. The neighboring stacks are inter­digitated through the shortened F⋯F, CH⋯F and CH⋯π contacts, forming a dense crystal structure.

## Abstract 2

In the title compound, mol­ecules are connected by Hg⋯O inter­actions. The 1:1 complexes pack in zigzag chains where they stack *via* two alternating stacking patterns, TPPM(cyclic trimeric perfluoro-*o*-phenyl­enemercury)–TPPM, and FLA(flavone)–FLA. The shortened F⋯F, CH⋯F and CH⋯π contacts consolidate the crystal structure.

## 1. Chemical context

Macrocyclic trimeric perfluoro-*o*-phenyl­enemercury [TPPM, (*o*-C_6_F_4_Hg)_3_] Lewis acid containing three Hg atoms in a planar nine-membered cycle has been used successfully in recent decades as a multidentate Lewis acid host. Numerous studies registered an excellent oxo- and thio­philicity of this strong electron acceptor manifested in its reactions with various anions and neutral Lewis bases to give complexes wherein the Lewis bases were easily cooperatively coordin­ated by multiple TPPM binding sites (King *et al.*, 2002*a* ▸,*b* ▸; Tikhonova *et al.*, 2005 ▸; Castañeda *et al.*, 2015 ▸, 2016 ▸; Loveday *et al.*, 2022 ▸). In particular, the oxophilicity of TPPM is well-documented, and the reported examples revealed variable molar ratios and packing arrangements of the TPPM acceptor and O-containing Lewis bases in the crystals (King *et al.*, 2002*a* ▸,*b* ▸; Tikhonova *et al.*, 2005 ▸; Castañeda *et al.*, 2016 ▸). The O⋯Hg coordination bonds were the primary inter­actions in those crystals that involved two or three Hg atoms of TPPM and the O-donor mol­ecules situated on one or both faces of the TPPM macrocycle.

Flavonoids are a family of polyphenolic compounds broadly produced in plants and found in the human diet. They are generally recognized as active pharmaceutical ingredients (API) with health-prolonging effects attributed to their anti­bacterial, anti­oxidant, anti­tumor, and anti-inflammatory properties (Cushnie & Lamb, 2005 ▸, 2011 ▸). Flavone (FLA), the simplest member of the class of flavones, can be tailored for significant modulation of its pharmacological activity and therefore serves as an effective scaffold in medicinal chemistry (Singh *et al.*, 2014 ▸). While the crystal chemistry of the TPPM acceptor is rather rich, only single examples have been reported for the crystalline forms of FLA (Waller *et al.*, 2003 ▸; van Tonder *et al.*, 2009*a* ▸,*b* ▸; Jiang *et al.*, 2014 ▸; Khandavilli *et al.*, 2018 ▸; Li *et al.*, 2019 ▸; He *et al.*, 2015 ▸). To fill this gap, we decided to cocrystallize FLA with TPPM. From a crystal-engineering perspective, it might be predicted that FLA, containing a benzo-γ-pyrone moiety bearing a phenyl substituent at position 2 and having a carbonyl group, would be predisposed for association with TPPM *via* oxophilic and stacking inter­actions. The crystal structure of the product of these inter­actions, the cocrystal (FLA)·(TPPM), is reported.
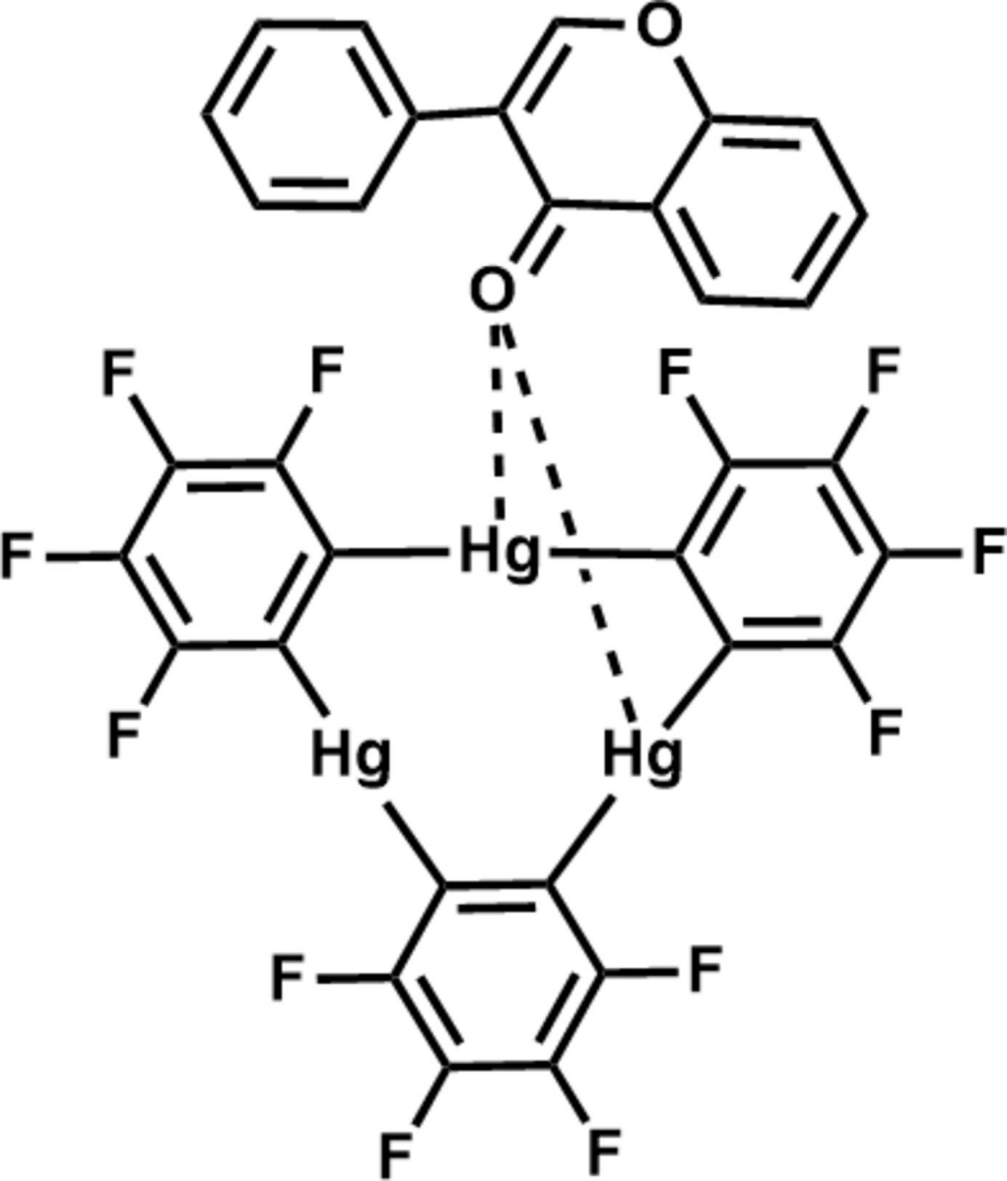


## 2. Structural commentary

**Figure 1:**
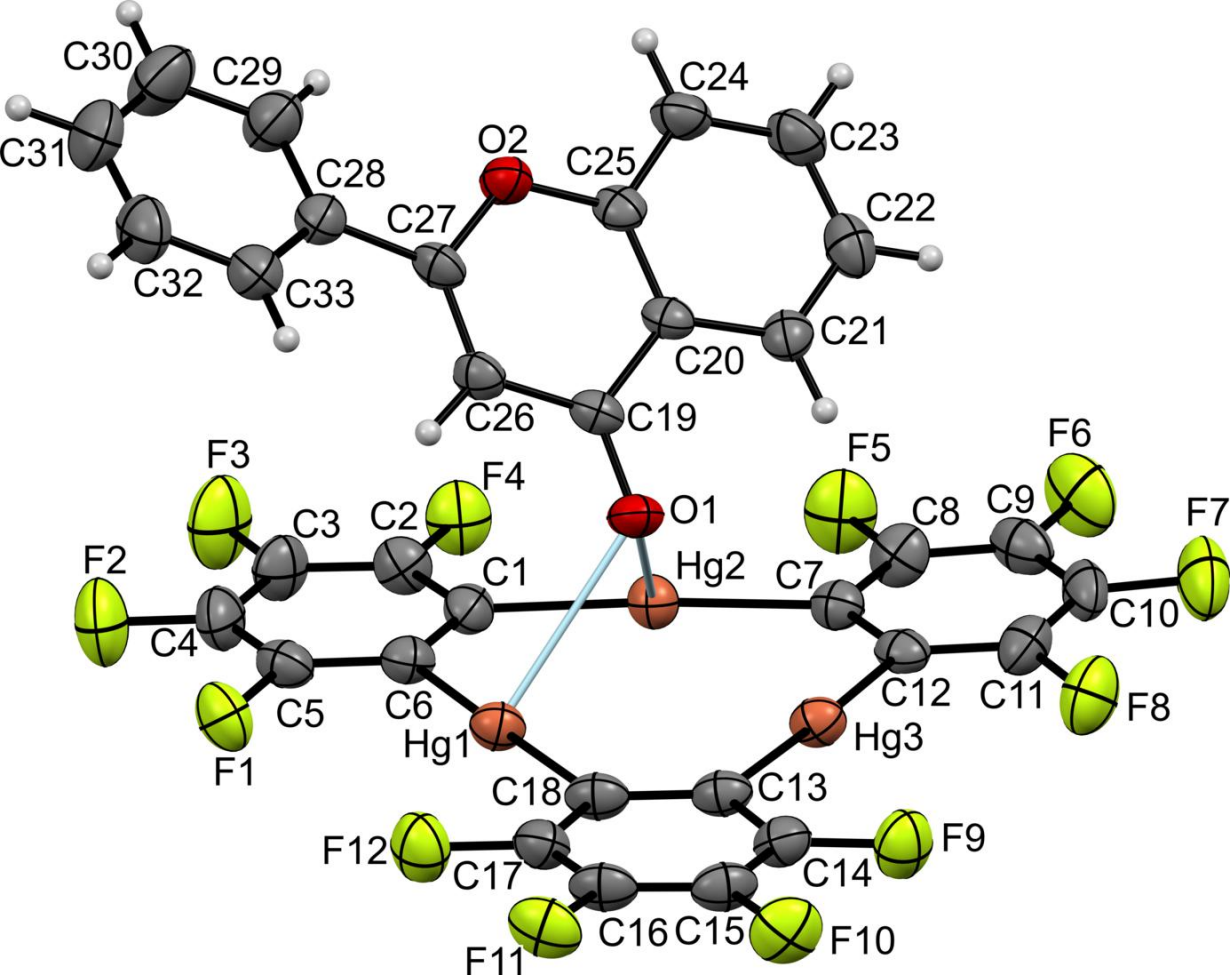
The mol­ecular structure of the title compound, with displacement ellipsoids drawn at the 50% probability level.

The title compound, (FLA)·(TPPM) in a 1:1 molar ratio, crystallizes in the monoclinic space group *P*2_1_/*n*. The asymmetric unit comprises one FLA and one TPPM mol­ecule (Fig. 1 ▸). The principal geometric parameters for both components are in good agreement with the literature values (Groom *et al.*, 2016 ▸). The components are held together *via* two Hg—O contacts, Hg1⋯O1 = 2.829 (3) and Hg2⋯O1 = 2.947 (4) Å, that are considerably shorter than the sum of the van der Waals radii of mercury (2.1 Å) and oxygen (1.5 Å) atoms (Batsanov, 2001 ▸; Yakovenko *et al.*, 2011 ▸). The Hg3⋯O1 distance is 3.097 (3) Å. Thus, an asymmetrical coordination of the FLA carbonyl oxygen atom, which is bonded to two Hg centers, is observed. The tilting angle between the virtually planar benzo-γ-pyrone residue (the r.m.s. deviation of the 11 fitted atoms is 0.0243 Å) and the mean plane of TPPM is 49.97 (5)°. The FLA mol­ecule has an angular shape indicated by the twisted angle between the benzo-γ-pyrone moiety and the anchored phenyl ring of 23.3 (2)°, contrary to the practically planar geometry of FLA in its pure form (WADRAV; Waller *et al.*, 2003 ▸) and in (η^6^-flavone)tri­carbonyl­chromium(0) (FUGBEP; van Tonder *et al.*, 2009*b* ▸).

## 3. Supra­molecular features

**Figure 2:**
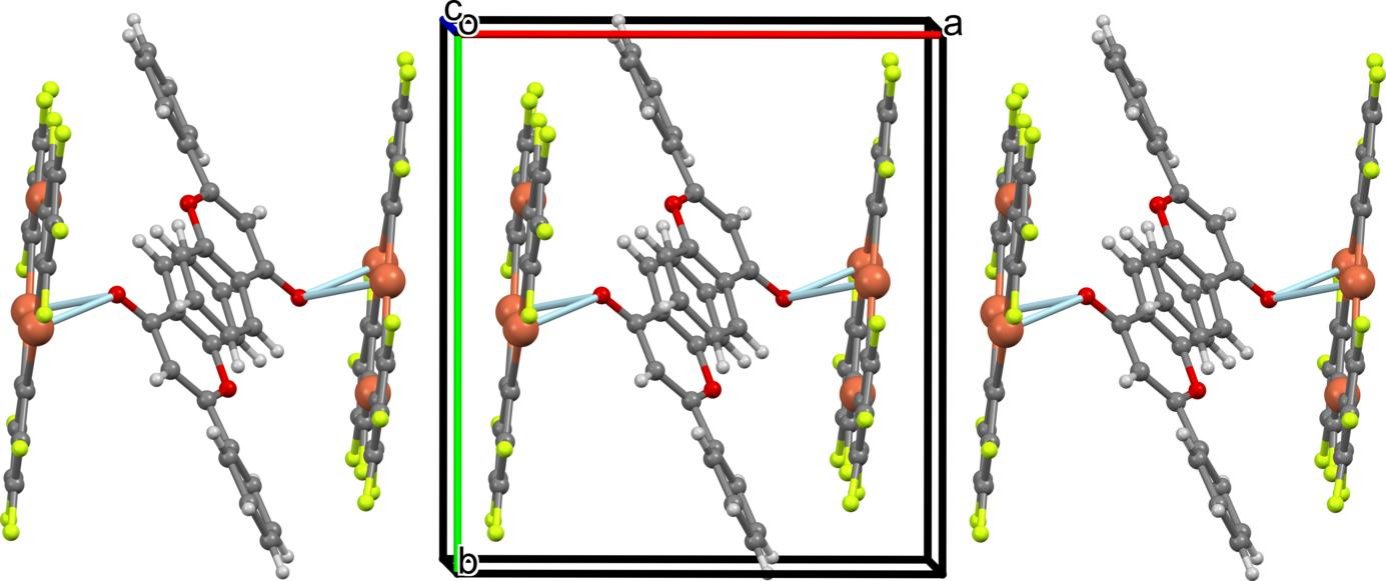
Supra­molecular chain in the title compound generated through alternation of TPPM–TPPM and FLA–FLA stacking patterns.

**Figure 3:**
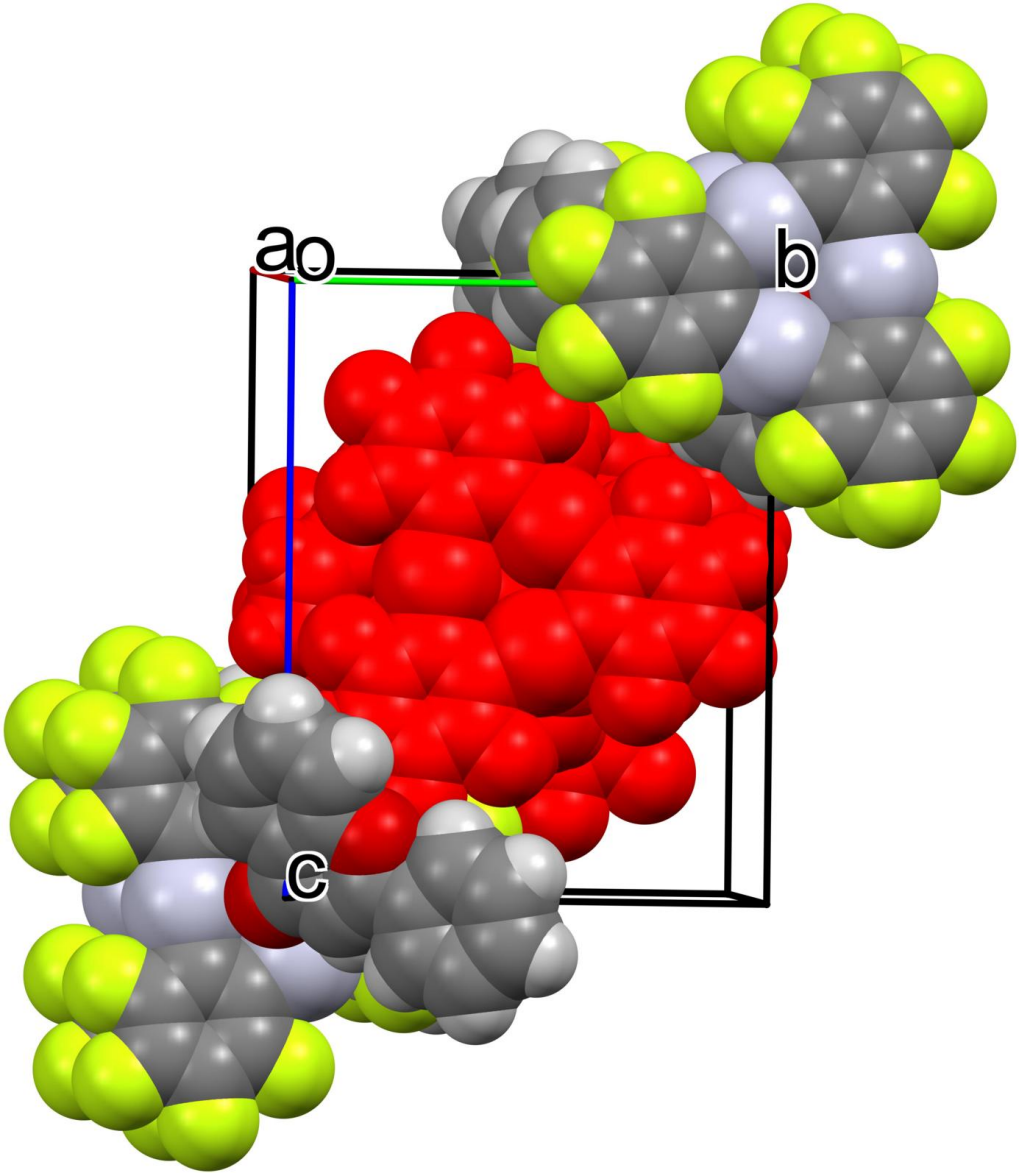
View of the crystal packing. The central stacking chain is shown in red.

The complexes pack in zigzag chains along the crystallographic *a* axis (Fig. 2 ▸), where they stack *via* two alternating TPPM–TPPM and FLA–FLA stacking patterns. The distance between the mean planes of the neighboring TPPM macrocycles in the stack is 3.445 (2) Å, and the macrocycles related by inversion are in a staggered conformation. The structure contains some inter­metallic Hg⋯Hg distances, shorter than sum of the van der Waals radii (Batsanov, 2001 ▸; Yakovenko *et al.*, 2011 ▸; Echeverría *et al.*, 2017 ▸). They include: Hg1⋯Hg2(2 − *x*, 1 − *y*, 1 − *z*) = 3.6305 (4) Å; Hg1⋯Hg3(2 − *x*, 1 − *y*, 1 − *z*) = 4.7390 (5) Å; Hg2⋯Hg2(2 − *x*, 1 − *y*, 1 − *z*) = 4.7514 (5) Å; Hg2⋯Hg3(2 − *x*, 1 − *y*, 1 − *z*) = 4.3423 (5) Å. The inter­planar separation between the two consecutive benzo-γ-pyrone moieties of FLA in the stack is equal to 3.328 (2) Å with only the pyrone rings overlapping, *Cg*(O2/C19–C27)⋯*Cg*(O2/C19–C27)(1 − *x*, 1 − *y*, 1 − *z*) = 3.996 (3) Å, slippage 2.211 Å. The neighboring stacks are inter­connected in an inter­digitated mode (Fig. 3 ▸) through the side F1⋯F11(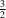 − *x*, *y* − 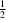, 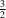 − *z*) 2.875 (5) Å, and CH⋯F shortened contacts, C24—H24⋯F1(*x* − 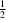, 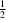 − *y*, *z* − 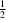) = 2.68 Å; C31—H31⋯F5(*x* − 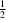, 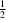 − *y*, 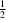 + *z*) = 2.62 Å; C32—H32⋯F5(*x* − 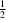, 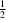 − *y*, 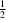 + *z*) = 2.50 Å; C30—H30⋯F11(*x* − 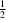, 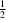 − *y*, *z* − 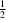) = 2.57 Å and C—H⋯π weak inter­actions (Table 1 ▸).

## 4. Database survey

A search in the Cambridge Structural Database (version 5.45, updated on 01/01/2024; Groom *et al.*, 2016 ▸) gave very few hits for FLA crystal forms. The pure form (WADRAV; Waller *et al.*, 2003 ▸) crystallizes in the *P*2_1_2_1_2_1_ space group with two crystallographically unique mol­ecules. In compound [Cr(FLA)(CO)_3_] (FUGBEP; van Tonder *et al.*, 2009*b* ▸), the Cr^III^ metal center coordinates the phenyl ring of FLA. In both cases, the FLA mol­ecule is significantly planar. In the case of the dapsone drug DAP–FLA 1:1 cocrystal (VOHKEK; Jiang *et al.*, 2014 ▸, *P*2_1_/*n* space group), the carbonyl group of FLA forms hydrogen-bonding inter­actions with the amino groups of DAP to form a tetra­meric aggregation. Furthermore, DAP–FLA was documented as another polymorph (Form B, VOHKEK01, *Fdd*2 space group) and another crystal form (RUHDOP, *P*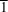 space group, He *et al.*, 2015 ▸) with a 1:2 ratio of component mol­ecules. In the drug cocrystal Naringenin–FLA (JILSIJ, 1:1 molar ratio; Khandavilli *et al.*, 2018 ▸) FLA mol­ecules are bridging between naringenin dimers *via* O—H⋯O inter­actions. The polymorphic diversity was also registered for the di­ethyl­stilbestrol–bis­(FLA) cocrystal (NOCTIL, *P*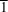 and NOCTIL01, *C*2/*c;* Li *et al.*, 2019 ▸). The high propensity of TPPM for carbonyl-containing compounds was demonstrated for: ethyl­acetate (CAMFIG; Tikhonova *et al.*, 2002 ▸; 3:1 donor–acceptor molar ratio), ethyl 3-oxo­butano­ate (KIRDIA; Tikhonova *et al.*, 2013 ▸), 4-(di­methyl­amino)­phen­yl­methanone (OGANIV; Fisher & Reinheimer, 2013 ▸; 1:1 molar ratio), acetone (PABLUA; King *et al.*, 2002*a* ▸,*b* ▸; 1:1 molar ratio), *N*,*N*-di­methyl­acetamide (XINMAI; Tikhonova *et al.*, 2001 ▸; 2:1 molar ratio), dimethyl formamide (XOJFEH; Baldamus *et al.*, 2002 ▸ and XOJFEH01; Tikhonova *et al.*, 2002 ▸; 2:1 molar ratio).

## 5. Synthesis and crystallization

FLA (11.212 mg, 0.05 mmol) was dissolved in 5 mL of DCM and TPPM (52.290 mg, 0.05 mmol) was added to the solution. The mixture was sonicated for 10 min at ambient conditions (room temperature), and after that the solution was transferred to a 15 mL test tube and covered with a cotton cap. In 10 days transparent colorless crystals (mass of crystals collected 54.205 mg, 0.0427 mmol, 85.35%) were obtained.

## 6. Refinement

Crystal data, data collection and structure refinement details are summarized in Table 2 ▸. All non-hydrogen atoms were refined in anisotropic approximation. The H atoms were refined as riding in idealized positions, with *U*_iso_(H) = 1.2*U*eq of the bearing C atom.

## Supplementary Material

Crystal structure: contains datablock(s) I. DOI: 10.1107/S2056989024005346/dj2077sup1.cif

Structure factors: contains datablock(s) I. DOI: 10.1107/S2056989024005346/dj2077Isup2.hkl

CCDC reference: 2360864

Additional supporting information:  crystallographic information; 3D view; checkCIF report

## Figures and Tables

**Table 1:** Hydrogen-bond geometry (Å, °)

*D*—H⋯*A*	*D*—H	H⋯*A*	*D*⋯*A*	*D*—H⋯*A*
C22—H22⋯F4^i^	0.95	2.54	3.486 (6)	173
C24—H24⋯F1^ii^	0.95	2.68	3.342 (6)	127
C30—H30⋯F11^ii^	0.95	2.57	3.473 (7)	160
C31—H31⋯F5^iii^	0.95	2.62	3.200 (8)	120
C32—H32⋯F5^iii^	0.95	2.50	3.149 (6)	126
C21—H21⋯C7	0.95	2.78	3.456 (7)	129
C24—H24⋯C16^iv^	0.95	2.82	3.621 (7)	142

Symmetry codes: (i) 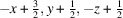; (ii) 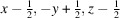; (iii) 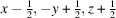; (iv) 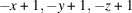.

**Table 2:** Experimental details

Crystal data	
Chemical formula	[Hg_3_(C_6_F_4_)_3_(C_15_H_10_O_2_)]
*M*_r_	1268.18
Crystal system, space group	Monoclinic, *P*2_1_/*n*
Temperature (K)	100
*a*, *b*, *c* (Å)	12.4181 (8), 13.8092 (8), 17.6498 (11)
β (°)	93.360 (2)
*V* (Å^3^)	3021.5 (3)
*Z*	4
Radiation type	Mo *K*α
μ (mm^−1^)	15.31
Crystal size (mm)	0.20 × 0.20 × 0.10

Data collection	
Diffractometer	Bruker SMART APEXII
Absorption correction	Multi-scan (*SADABS*; Krause *et al.*, 2015 ▸)
*T*_min_, *T*_max_	0.372, 0.746
No. of measured, independent and observed [*I* > 2σ(*I*)] reflections	38196, 5894, 5069
*R*_int_	0.039
(sin θ/λ)_max_ (Å^−1^)	0.617

Refinement	
*R*[*F*^2^ > 2σ(*F*^2^)], *wR*(*F*^2^), *S*	0.023, 0.052, 1.05
No. of reflections	5894
No. of parameters	451
H-atom treatment	H-atom parameters constrained
Δρ_max_, Δρ_min_ (e Å^−3^)	0.64, −1.19

Computer programs: *APEX2* (Bruker, 2019 ▸), *SAINT-Plus* (Bruker, 2020 ▸), *SHELXT* (Sheldrick, 2015*a* ▸), *SHELXL* (Sheldrick, 2015*b* ▸) and *OLEX2* (Dolomanov *et al.*, 2009 ▸).

## supplementary crystallographic information

### Tris(µ2-perfluoro-o-phenylene)(µ2-3-phenyl-4H-chromen-4-one)-triangulo-trimercury Crystal data

**Table:**

[Hg_3_(C_6_F_4_)_3_(C_15_H_10_O_2_)]	*F*(000) = 2288
*M**_r_* = 1268.18	*D*_x_ = 2.788 Mg m^−^^3^
Monoclinic, *P*2_1_/*n*	Mo *K*α radiation, λ = 0.71073 Å
*a* = 12.4181 (8) Å	Cell parameters from 9132 reflections
*b* = 13.8092 (8) Å	θ = 2.2–26.7°
*c* = 17.6498 (11) Å	µ = 15.31 mm^−^^1^
β = 93.360 (2)°	*T* = 100 K
*V* = 3021.5 (3) Å^3^	Plate, clear colourless
*Z* = 4	0.20 × 0.20 × 0.10 mm

### Tris(µ2-perfluoro-o-phenylene)(µ2-3-phenyl-4H-chromen-4-one)-triangulo-trimercury Data collection

**Table:**

Bruker SMART APEXII diffractometer	5894 independent reflections
Radiation source: sealed X-ray tube, EIGENMANN GmbH	5069 reflections with *I* > 2σ(*I*)
Graphite monochromator	*R*_int_ = 0.039
Detector resolution: 7.9 pixels mm^-1^	θ_max_ = 26.0°, θ_min_ = 1.9°
ω and φ scans	*h* = −15→15
Absorption correction: multi-scan (SADABS; Krause *et al.*, 2015)	*k* = −15→17
*T*_min_ = 0.372, *T*_max_ = 0.746	*l* = −21→16
38196 measured reflections	

### Tris(µ2-perfluoro-o-phenylene)(µ2-3-phenyl-4H-chromen-4-one)-triangulo-trimercury Refinement

**Table:**

Refinement on *F*^2^	Primary atom site location: structure-invariant direct methods
Least-squares matrix: full	Secondary atom site location: difference Fourier map
*R*[*F*^2^ > 2σ(*F*^2^)] = 0.023	Hydrogen site location: inferred from neighbouring sites
*wR*(*F*^2^) = 0.052	H-atom parameters constrained
*S* = 1.05	*w* = 1/[σ^2^(*F*_o_^2^) + (0.0252*P*)^2^ + 1.6597*P*] where *P* = (*F*_o_^2^ + 2*F*_c_^2^)/3
5894 reflections	(Δ/σ)_max_ = 0.003
451 parameters	Δρ_max_ = 0.64 e Å^−^^3^
0 restraints	Δρ_min_ = −1.19 e Å^−^^3^

### Tris(µ2-perfluoro-o-phenylene)(µ2-3-phenyl-4H-chromen-4-one)-triangulo-trimercury Special details

**Table:**

Geometry. All esds (except the esd in the dihedral angle between two l.s. planes) are estimated using the full covariance matrix. The cell esds are taken into account individually in the estimation of esds in distances, angles and torsion angles; correlations between esds in cell parameters are only used when they are defined by crystal symmetry. An approximate (isotropic) treatment of cell esds is used for estimating esds involving l.s. planes.

### Tris(µ2-perfluoro-o-phenylene)(µ2-3-phenyl-4H-chromen-4-one)-triangulo-trimercury Fractional atomic coordinates and isotropic or equivalent isotropic displacement parameters (Å^2^)

**Table:**

	*x*	*y*	*z*	*U*_iso_*/*U*_eq_	
Hg1	0.85812 (2)	0.44283 (2)	0.60352 (2)	0.03572 (6)	
Hg2	0.86702 (2)	0.46865 (2)	0.40075 (2)	0.03547 (6)	
Hg3	0.84037 (2)	0.68004 (2)	0.51822 (2)	0.03658 (6)	
F1	0.8927 (3)	0.2205 (2)	0.63984 (17)	0.0536 (8)	
F2	0.9080 (3)	0.0628 (2)	0.5516 (2)	0.0729 (11)	
F3	0.9084 (3)	0.0823 (2)	0.3984 (2)	0.0777 (12)	
F4	0.8941 (3)	0.2590 (2)	0.33427 (17)	0.0561 (9)	
F5	0.8718 (3)	0.5418 (2)	0.23224 (18)	0.0688 (10)	
F6	0.8452 (3)	0.7185 (3)	0.16752 (18)	0.0751 (11)	
F7	0.8183 (3)	0.8760 (2)	0.2553 (2)	0.0702 (10)	
F8	0.8354 (3)	0.8603 (2)	0.4079 (2)	0.0646 (10)	
F9	0.8232 (3)	0.8296 (2)	0.65175 (18)	0.0565 (9)	
F10	0.8092 (3)	0.8092 (2)	0.80319 (17)	0.0571 (9)	
F11	0.8165 (3)	0.6306 (2)	0.86695 (16)	0.0552 (8)	
F12	0.8461 (3)	0.4725 (2)	0.78054 (18)	0.0583 (9)	
O1	0.6883 (3)	0.5027 (2)	0.50025 (18)	0.0383 (8)	
O2	0.4563 (3)	0.3256 (2)	0.40217 (19)	0.0379 (8)	
C1	0.8790 (4)	0.3342 (3)	0.4535 (3)	0.0365 (12)	
C2	0.8910 (4)	0.2518 (4)	0.4107 (3)	0.0404 (12)	
C3	0.8998 (5)	0.1606 (4)	0.4419 (3)	0.0470 (14)	
C4	0.8998 (5)	0.1504 (4)	0.5191 (3)	0.0491 (14)	
C5	0.8890 (4)	0.2323 (4)	0.5636 (3)	0.0395 (12)	
C6	0.8776 (4)	0.3242 (3)	0.5331 (3)	0.0345 (11)	
C7	0.8593 (4)	0.6074 (3)	0.3557 (3)	0.0352 (11)	
C8	0.8596 (4)	0.6197 (4)	0.2781 (3)	0.0438 (13)	
C9	0.8465 (4)	0.7088 (4)	0.2435 (3)	0.0469 (14)	
C10	0.8346 (4)	0.7894 (4)	0.2882 (3)	0.0451 (14)	
C11	0.8394 (4)	0.7791 (4)	0.3655 (3)	0.0443 (13)	
C12	0.8482 (4)	0.6903 (4)	0.4009 (3)	0.0348 (11)	
C13	0.8368 (4)	0.6593 (4)	0.6347 (3)	0.0359 (11)	
C14	0.8278 (4)	0.7389 (4)	0.6815 (3)	0.0398 (12)	
C15	0.8211 (4)	0.7302 (4)	0.7593 (3)	0.0411 (13)	
C16	0.8264 (4)	0.6408 (4)	0.7915 (3)	0.0422 (13)	
C17	0.8405 (4)	0.5607 (4)	0.7459 (3)	0.0406 (12)	
C18	0.8448 (4)	0.5670 (4)	0.6683 (3)	0.0391 (12)	
C19	0.6156 (4)	0.4494 (3)	0.4705 (3)	0.0304 (10)	
C20	0.5632 (4)	0.4696 (3)	0.3954 (3)	0.0307 (10)	
C21	0.5875 (4)	0.5526 (3)	0.3525 (3)	0.0361 (12)	
H21	0.6385	0.5985	0.3727	0.043*	
C22	0.5381 (4)	0.5673 (4)	0.2823 (3)	0.0463 (14)	
H22	0.5544	0.6234	0.2541	0.056*	
C23	0.4647 (5)	0.5010 (4)	0.2524 (3)	0.0479 (14)	
H23	0.4318	0.5114	0.2031	0.058*	
C24	0.4380 (4)	0.4199 (4)	0.2926 (3)	0.0431 (13)	
H24	0.3870	0.3744	0.2719	0.052*	
C25	0.4880 (4)	0.4066 (3)	0.3643 (3)	0.0328 (11)	
C26	0.5780 (4)	0.3633 (3)	0.5056 (3)	0.0335 (11)	
H26	0.6081	0.3460	0.5545	0.040*	
C27	0.5022 (4)	0.3063 (3)	0.4723 (3)	0.0346 (11)	
C28	0.4558 (4)	0.2176 (3)	0.5049 (3)	0.0378 (12)	
C29	0.4084 (5)	0.1470 (4)	0.4570 (3)	0.0544 (15)	
H29	0.4040	0.1560	0.4036	0.065*	
C30	0.3682 (6)	0.0639 (4)	0.4884 (4)	0.0685 (19)	
H30	0.3353	0.0161	0.4560	0.082*	
C31	0.3747 (5)	0.0488 (4)	0.5655 (4)	0.0621 (17)	
H31	0.3485	−0.0096	0.5861	0.075*	
C32	0.4189 (5)	0.1186 (4)	0.6118 (3)	0.0548 (15)	
H32	0.4214	0.1097	0.6653	0.066*	
C33	0.4605 (4)	0.2024 (4)	0.5820 (3)	0.0451 (13)	
H33	0.4926	0.2499	0.6151	0.054*	

### Tris(µ2-perfluoro-o-phenylene)(µ2-3-phenyl-4H-chromen-4-one)-triangulo-trimercury Atomic displacement parameters (Å^2^)

**Table:**

	*U*^11^	*U*^22^	*U*^33^	*U*^12^	*U*^13^	*U*^23^
Hg1	0.03653 (11)	0.03970 (11)	0.03063 (12)	−0.00036 (8)	−0.00069 (9)	−0.00388 (8)
Hg2	0.03836 (12)	0.03602 (11)	0.03231 (12)	0.00085 (8)	0.00439 (9)	−0.00019 (8)
Hg3	0.03959 (12)	0.04065 (11)	0.02955 (12)	−0.00449 (8)	0.00228 (9)	−0.00388 (8)
F1	0.064 (2)	0.0567 (19)	0.0405 (19)	0.0088 (16)	0.0019 (16)	0.0120 (15)
F2	0.102 (3)	0.0436 (19)	0.074 (3)	0.0153 (18)	0.010 (2)	0.0131 (17)
F3	0.122 (3)	0.0389 (18)	0.074 (3)	0.012 (2)	0.017 (2)	−0.0130 (17)
F4	0.077 (2)	0.0521 (19)	0.0403 (19)	0.0064 (17)	0.0113 (17)	−0.0080 (14)
F5	0.106 (3)	0.064 (2)	0.038 (2)	0.003 (2)	0.015 (2)	−0.0125 (16)
F6	0.096 (3)	0.094 (3)	0.036 (2)	0.001 (2)	0.0106 (19)	0.0207 (18)
F7	0.079 (3)	0.054 (2)	0.079 (3)	0.0043 (18)	0.017 (2)	0.0299 (18)
F8	0.080 (2)	0.0400 (18)	0.075 (2)	−0.0009 (17)	0.015 (2)	−0.0028 (16)
F9	0.081 (2)	0.0413 (18)	0.047 (2)	−0.0048 (16)	−0.0001 (18)	−0.0047 (14)
F10	0.072 (2)	0.057 (2)	0.0425 (19)	−0.0012 (16)	0.0048 (17)	−0.0203 (15)
F11	0.061 (2)	0.076 (2)	0.0272 (17)	0.0027 (17)	−0.0009 (15)	−0.0049 (15)
F12	0.080 (2)	0.0523 (19)	0.0422 (19)	0.0006 (17)	−0.0017 (18)	0.0052 (15)
O1	0.0331 (19)	0.043 (2)	0.038 (2)	−0.0075 (16)	−0.0073 (16)	−0.0013 (16)
O2	0.041 (2)	0.0398 (19)	0.032 (2)	−0.0084 (15)	−0.0041 (16)	0.0024 (14)
C1	0.031 (3)	0.034 (3)	0.045 (3)	0.003 (2)	0.002 (2)	0.001 (2)
C2	0.046 (3)	0.044 (3)	0.032 (3)	0.004 (2)	0.005 (2)	−0.004 (2)
C3	0.061 (4)	0.033 (3)	0.047 (4)	0.003 (3)	0.006 (3)	−0.010 (2)
C4	0.052 (3)	0.033 (3)	0.062 (4)	0.008 (3)	0.001 (3)	0.005 (3)
C5	0.035 (3)	0.051 (3)	0.032 (3)	0.000 (2)	−0.003 (2)	0.003 (2)
C6	0.031 (3)	0.038 (3)	0.035 (3)	0.001 (2)	0.000 (2)	−0.003 (2)
C7	0.032 (3)	0.042 (3)	0.032 (3)	−0.002 (2)	0.004 (2)	0.001 (2)
C8	0.050 (3)	0.047 (3)	0.035 (3)	−0.003 (2)	0.014 (3)	−0.003 (2)
C9	0.042 (3)	0.067 (4)	0.033 (3)	−0.001 (3)	0.003 (3)	0.011 (3)
C10	0.040 (3)	0.042 (3)	0.053 (4)	−0.001 (2)	0.007 (3)	0.021 (3)
C11	0.043 (3)	0.035 (3)	0.056 (4)	−0.006 (2)	0.011 (3)	−0.002 (3)
C12	0.025 (2)	0.046 (3)	0.034 (3)	−0.003 (2)	0.001 (2)	0.003 (2)
C13	0.032 (3)	0.047 (3)	0.029 (3)	−0.006 (2)	0.002 (2)	−0.006 (2)
C14	0.038 (3)	0.044 (3)	0.037 (3)	−0.007 (2)	0.001 (2)	−0.003 (2)
C15	0.038 (3)	0.047 (3)	0.038 (3)	−0.003 (2)	0.001 (2)	−0.017 (2)
C16	0.040 (3)	0.060 (3)	0.026 (3)	−0.004 (3)	−0.002 (2)	−0.010 (2)
C17	0.038 (3)	0.045 (3)	0.038 (3)	−0.002 (2)	−0.005 (2)	0.000 (2)
C18	0.036 (3)	0.052 (3)	0.029 (3)	−0.004 (2)	−0.001 (2)	−0.008 (2)
C19	0.026 (2)	0.039 (3)	0.026 (3)	0.004 (2)	−0.001 (2)	−0.003 (2)
C20	0.023 (2)	0.040 (3)	0.029 (3)	0.001 (2)	0.000 (2)	0.000 (2)
C21	0.031 (3)	0.037 (3)	0.040 (3)	0.000 (2)	0.005 (2)	0.003 (2)
C22	0.051 (3)	0.046 (3)	0.042 (3)	0.006 (3)	0.006 (3)	0.013 (2)
C23	0.052 (4)	0.058 (4)	0.032 (3)	0.001 (3)	−0.007 (3)	0.006 (3)
C24	0.040 (3)	0.054 (3)	0.034 (3)	−0.006 (2)	−0.008 (2)	0.000 (2)
C25	0.024 (2)	0.042 (3)	0.033 (3)	0.000 (2)	0.004 (2)	0.003 (2)
C26	0.035 (3)	0.042 (3)	0.024 (3)	−0.001 (2)	−0.001 (2)	0.003 (2)
C27	0.036 (3)	0.043 (3)	0.024 (3)	0.002 (2)	−0.004 (2)	0.003 (2)
C28	0.036 (3)	0.035 (3)	0.043 (3)	−0.001 (2)	0.001 (2)	0.002 (2)
C29	0.065 (4)	0.050 (3)	0.049 (4)	−0.016 (3)	0.001 (3)	0.001 (3)
C30	0.084 (5)	0.053 (4)	0.069 (5)	−0.024 (3)	0.005 (4)	−0.008 (3)
C31	0.068 (4)	0.049 (4)	0.071 (5)	−0.013 (3)	0.007 (4)	0.015 (3)
C32	0.060 (4)	0.055 (4)	0.049 (4)	−0.004 (3)	0.007 (3)	0.018 (3)
C33	0.042 (3)	0.045 (3)	0.048 (4)	−0.001 (2)	−0.003 (3)	0.006 (2)

### Tris(µ2-perfluoro-o-phenylene)(µ2-3-phenyl-4H-chromen-4-one)-triangulo-trimercury Geometric parameters (Å, º)

**Table:**

Hg1—C18	2.072 (5)	C13—C14	1.383 (7)
Hg1—C6	2.079 (5)	C13—C18	1.407 (7)
Hg2—C7	2.074 (5)	C14—C15	1.387 (7)
Hg2—C1	2.079 (5)	C15—C16	1.358 (7)
Hg3—C13	2.079 (5)	C16—C17	1.385 (7)
Hg3—C12	2.084 (5)	C17—C18	1.377 (7)
F1—C5	1.355 (6)	C19—C26	1.432 (6)
F2—C4	1.338 (6)	C19—C20	1.468 (6)
F3—C3	1.335 (6)	C20—C25	1.368 (6)
F4—C2	1.356 (6)	C20—C21	1.416 (6)
F5—C8	1.359 (6)	C21—C22	1.364 (7)
F6—C9	1.347 (6)	C21—H21	0.9500
F7—C10	1.339 (6)	C22—C23	1.374 (8)
F8—C11	1.351 (6)	C22—H22	0.9500
F9—C14	1.358 (6)	C23—C24	1.377 (7)
F10—C15	1.352 (5)	C23—H23	0.9500
F11—C16	1.352 (6)	C24—C25	1.388 (7)
F12—C17	1.363 (6)	C24—H24	0.9500
O1—C19	1.256 (5)	C26—C27	1.337 (7)
O2—C27	1.358 (6)	C26—H26	0.9500
O2—C25	1.373 (5)	C27—C28	1.484 (6)
C1—C2	1.378 (6)	C28—C33	1.374 (7)
C1—C6	1.413 (7)	C28—C29	1.397 (7)
C2—C3	1.376 (7)	C29—C30	1.380 (8)
C3—C4	1.371 (8)	C29—H29	0.9500
C4—C5	1.388 (7)	C30—C31	1.374 (9)
C5—C6	1.382 (7)	C30—H30	0.9500
C7—C8	1.381 (7)	C31—C32	1.358 (8)
C7—C12	1.406 (7)	C31—H31	0.9500
C8—C9	1.379 (7)	C32—C33	1.385 (7)
C9—C10	1.376 (8)	C32—H32	0.9500
C10—C11	1.370 (8)	C33—H33	0.9500
C11—C12	1.377 (7)		
			
C18—Hg1—C6	175.85 (19)	F12—C17—C18	119.9 (4)
C7—Hg2—C1	175.8 (2)	F12—C17—C16	117.3 (5)
C13—Hg3—C12	175.7 (2)	C18—C17—C16	122.8 (5)
C27—O2—C25	119.1 (4)	C17—C18—C13	118.0 (4)
C2—C1—C6	118.2 (4)	C17—C18—Hg1	120.4 (4)
C2—C1—Hg2	120.0 (4)	C13—C18—Hg1	121.5 (4)
C6—C1—Hg2	121.8 (3)	O1—C19—C26	123.4 (4)
F4—C2—C3	117.3 (4)	O1—C19—C20	122.4 (4)
F4—C2—C1	119.6 (4)	C26—C19—C20	114.2 (4)
C3—C2—C1	123.1 (5)	C25—C20—C21	117.5 (4)
F3—C3—C4	119.6 (5)	C25—C20—C19	119.8 (4)
F3—C3—C2	121.3 (5)	C21—C20—C19	122.7 (4)
C4—C3—C2	119.2 (5)	C22—C21—C20	120.4 (5)
F2—C4—C3	120.9 (5)	C22—C21—H21	119.8
F2—C4—C5	120.2 (5)	C20—C21—H21	119.8
C3—C4—C5	118.9 (5)	C21—C22—C23	120.2 (5)
F1—C5—C6	119.7 (4)	C21—C22—H22	119.9
F1—C5—C4	117.6 (5)	C23—C22—H22	119.9
C6—C5—C4	122.7 (5)	C22—C23—C24	121.2 (5)
C5—C6—C1	118.0 (4)	C22—C23—H23	119.4
C5—C6—Hg1	120.2 (4)	C24—C23—H23	119.4
C1—C6—Hg1	121.8 (3)	C23—C24—C25	117.9 (5)
C8—C7—C12	117.9 (4)	C23—C24—H24	121.0
C8—C7—Hg2	119.4 (4)	C25—C24—H24	121.0
C12—C7—Hg2	122.6 (3)	C20—C25—O2	122.1 (4)
F5—C8—C9	117.2 (5)	C20—C25—C24	122.7 (4)
F5—C8—C7	120.0 (5)	O2—C25—C24	115.2 (4)
C9—C8—C7	122.8 (5)	C27—C26—C19	122.5 (5)
F6—C9—C10	119.6 (5)	C27—C26—H26	118.7
F6—C9—C8	121.5 (5)	C19—C26—H26	118.7
C10—C9—C8	118.8 (5)	C26—C27—O2	122.2 (4)
F7—C10—C11	121.4 (5)	C26—C27—C28	126.5 (5)
F7—C10—C9	119.5 (5)	O2—C27—C28	111.3 (4)
C11—C10—C9	119.1 (5)	C33—C28—C29	118.9 (5)
F8—C11—C10	117.7 (5)	C33—C28—C27	120.9 (5)
F8—C11—C12	119.5 (5)	C29—C28—C27	120.1 (5)
C10—C11—C12	122.8 (5)	C30—C29—C28	119.2 (6)
C11—C12—C7	118.4 (5)	C30—C29—H29	120.4
C11—C12—Hg3	120.2 (4)	C28—C29—H29	120.4
C7—C12—Hg3	121.3 (3)	C31—C30—C29	121.5 (6)
C14—C13—C18	118.3 (4)	C31—C30—H30	119.3
C14—C13—Hg3	119.2 (4)	C29—C30—H30	119.3
C18—C13—Hg3	122.5 (3)	C32—C31—C30	119.1 (5)
F9—C14—C13	120.4 (4)	C32—C31—H31	120.4
F9—C14—C15	117.3 (4)	C30—C31—H31	120.4
C13—C14—C15	122.3 (5)	C31—C32—C33	120.7 (6)
F10—C15—C16	119.9 (5)	C31—C32—H32	119.6
F10—C15—C14	120.8 (5)	C33—C32—H32	119.6
C16—C15—C14	119.2 (4)	C28—C33—C32	120.6 (5)
F11—C16—C15	120.1 (4)	C28—C33—H33	119.7
F11—C16—C17	120.7 (5)	C32—C33—H33	119.7
C15—C16—C17	119.2 (5)		

### Tris(µ2-perfluoro-o-phenylene)(µ2-3-phenyl-4H-chromen-4-one)-triangulo-trimercury Hydrogen-bond geometry (Å, º)

**Table:**

*D*—H···*A*	*D*—H	H···*A*	*D*···*A*	*D*—H···*A*
C22—H22···F4^i^	0.95	2.54	3.486 (6)	173
C24—H24···F1^ii^	0.95	2.68	3.342 (6)	127
C30—H30···F11^ii^	0.95	2.57	3.473 (7)	160
C31—H31···F5^iii^	0.95	2.62	3.200 (8)	120
C32—H32···F5^iii^	0.95	2.50	3.149 (6)	126
C21—H21···C7	0.95	2.78	3.456 (7)	129
C24—H24···C16^iv^	0.95	2.82	3.621 (7)	142

Symmetry codes: (i) −*x*+3/2, *y*+1/2, −*z*+1/2; (ii) *x*−1/2, −*y*+1/2, *z*−1/2; (iii) *x*−1/2, −*y*+1/2, *z*+1/2; (iv) −*x*+1, −*y*+1, −*z*+1.
